# Complementary feeding practice and associated factors among internally displaced mothers of children aged 6–23 months in Amhara region, Northwest Ethiopia: a cross-sectional study

**DOI:** 10.1186/s12887-021-03050-y

**Published:** 2021-12-20

**Authors:** Alex Yeshaneh, Meron Zebene, Molla Gashu, Haimanot Abebe, Habtemariam Abate

**Affiliations:** 1 Department of Midwifery, College of Medicine and Health Science, Wolkite University, Wolkite, Ethiopia; 2 Ethiopian Federal Ministry of Health, Addis Ababa, Ethiopia; 3 Department of Public Health, College of Medicine and Health Sciences, Wolkite University, Wolkite, Ethiopia; 4 Federal Food, Medicine, and Health care Administration and Control Authority, Addis Ababa, Ethiopia

**Keywords:** Complementary feeding practice, Internally displaced people, Children 6–23 months, Gendwuha district, Ethiopia

## Abstract

**Background:**

Internally displaced populations are susceptible to food deprivation. Specifically, children aged 6–23 are commonly vulnerable to poor complementary feeding. Proper complementary feeding is of paramount importance to the healthy growth and survival of a children. Therefore, this study aimed to assess the level of appropriate complementary feeding practices and associated factors among internally displaced children aged 6–23 months in northwest Ethiopia, 2020.

**Methods:**

A community-based cross-sectional study was conducted from June to July 2020 among 264 internally displaced mothers of children 6–23 months in northwest Ethiopia. A systematic random sampling technique was used to reach the study subjects and data were collected using a structured and pre-tested interviewer-administered questionnaire. Data were entered into the Epi Data version 4.1 and analyzed using SPSS version 23. Binary and multivariable analyses with a 95% confidence level were performed. In the final model, variables with *P* < 0.05 were considered statistically significant.

**Results:**

The overall level of appropriate complementary feeding practice was 26.8%. Only 14% of the mothers provided a diversified diet for their 6–23 months children. Child aged 6–11 months (AOR = 0.11, 95%; CI: 0.04–0.27), 12–17 months (AOR = 0.35, 95%; 95% CI: 0.17–0.70) and not having harmful culture on complementary feeding (AOR = 2.04; 95% CI: 1.06–3.96) were independent predictors of appropriate complementary feeding practices.

**Conclusion:**

The level of appropriate complementary feeding practice was found to be low, which would have negative implications on the health and nutritional status of infants and young children. Additional rations for breastfeeding mothers and children aged 6–23 months at refugee camps and nutritional counseling on child feeding practices are recommended.

**Supplementary Information:**

The online version contains supplementary material available at 10.1186/s12887-021-03050-y.

## Introduction

An internally displaced person (IDP) is someone who is enforced to leave her/his home due to conflict or other different factors and remains within his/her country’s borders but does not fulfill the legal definitions of a refugee. Sub-Saharan Africa accounts for only 14% of the world’s population, but almost half of the new conflict displacement occurred in the region. In 2020, about 1.7 million new displacements were associated with conflict and violence in Ethiopia. Of these, 67,547 people were fled by conflict in Amhara Ethiopia in July [1, 2].

Internally displaced mothers are often vulnerable and more regularly deprived of food, water, health care, and other essentials. Children are one of the more vulnerable groups in which displacement has to disrupt feeding practices and long-term effects on development [2, 3].

Complementary feeding is a transition from exclusive breastfeeding to family foods that typically covers the period from 6 to 24 months of age. It is considered appropriate when it is timely, adequate, safe, and properly fed. The period of 6–23 months is a critical period of growth during which nutrient deficiencies and illnesses contribute globally to higher rates of undernutrition. Appropriate complementary feeding depends not only on the availability of foods but also on the feeding practices of caregivers [1, 4].

Globally, only 64.5% of infants at 6–8 months of age are fed solid, semisolid, or soft foods, with the lowest rates in South Asia, at 53.5%, and highest in Latin America and the Caribbean (LAC), at 83.1%. In West and Central Africa, East and South Africa, and South Asia, the rates of MMF are less than 50% and MDD is less than 25%. Rates for both indicators are highest in East Asia and the Pacific and LAC where about three in four children were recieving MMF and MDD. According to the Ethiopian demographic health survey (EDHS) 2016, only 7% of children aged 6–23 months met the minimum acceptable diet (MAD) and only 14% of children were fed from the appropriate number of food groups (MDD) and 45% of children were fed the MMF [2.4].

Several successful strategies have been developed to improve the appropriate complementary feeding practices of low and middle-income countries, where practical difficulties can limit the process. These challenges could worsen among internally displaced people. Scientific evidence indicates that various appropriate complementary feeding practices such as the timely introduction of complementary food, proper feeding frequency, and high dietary diversity of complementary foods have been shown to have numerous positive effects on children’s health [1, 3, 5]. Studies on appropriate complementary feeding practices among IDPs in our country are very limited. Therefore, this study aimed to assess the level of appropriate complementary feeding practices and associated factors among internally displaced mothers of children 6–23 months.

## Methods

### Study setting and design

Gendewuha is a district located in the west Gondar Amhara region, 160 km from Gondar city. It has a total host population of 29,874 and 6000 IDPs. From the host population, 14,105 and 15,769 were men and women respectively. Approximaly, 8724 are children of aged less than 14 years. Of the IDPs, 642 are known to be mothers of children aged 6–23 months who live collectively in the Gendewuha IDP camp Amhara region, northwest Ethiopia. A community-based cross-sectional study design was conducted from June to July 2020.

### Source and study population

The source population consisted of all internally displaced mothers of children aged 6–23 months who were living in the Amhara region, northwest Ethiopia. The study population included internally displaced mothers of children 6–23 months from those living in Gendewuha camp, northwest, Ethiopia.

### Exclusion criteria

Mothers of children aged 6–23 months who visited internally displaced populations during the data collection period were excluded from the study.

### Sampling size determination and sampling procedure

The required sample size was calculated using OpenEpi statistics software version 3.03 by considering the assumptions (80% power of the study, 95% confidence level, 1:1 ratio) and the prevalence of complementary feeding practice (19.4%) from a previous study [6]. By adding a 10% non-response rate, the final sample size was 264 study participants.

A systematic random sampling technique was used to obtain to reach the final study units. Since internally displaced populations are not stable, a survey assessment was performed to count the number of IDP mothers of children found at the time of the data collection, and tent numbers were given and used as a sampling frame. “K” was calculated by dividing the total 642 mothers of children of age 6–23 months present at the site at the time of the data collection period with the sample size, 264, and was found to be “2”. Households with mothers of children aged 6–23 months were recruited in every other household. Households with selected mothers of children aged 6–23 months were identified based on the tent numbers given during the quick survey assessment before data collection. In a situation where there was more than one mother of children aged 6–23 months, the lottery method was used to select one of them. Likewise, whenever a mother had more than one child of age 6–23 months of age, the lottery method was used to select one of the children.

### Data collection tools and procedures

Data were collected using a questionnaire that was partially adapted, structured, interviewer-administered, and closed-ended questionnaires. The tool has 4 sections including dietary diversity inquiry for infants and young children which was adopted from a food and nutrition technical assistant (FANTA). A 24-h dietary recall method, which is the most widely used time period for collecting dietary information, was used to collect dietary information. The data collectors were six diploma nurses and one supervisor who is a BSC nurse. Two days of training were given to the data collectors and the supervisor. The questionnaire was translated to the local language [Amharic] by a language translator and retranslated back in to English to check its consistency. The questionnaire was pre-tested on 5% of the study population before the actual data collection at other sites with a similar population (Chillga IDP site) and corrections were made on some of the ambiguities in the questionnaire. Those mothers who were absent for the first visit were re-visited for two additional visits on different days and were considered non-responders after an absence for three visits. Data completeness and consistency were checked by the supervisor as well as by the principal investigator on a daily basis.

### Study variables

#### Dependent variable

Complementary feeding practices.

#### Independent variables

The independent variables of the study were: Socioeconomic and demographic characteristics such as child age, child sex, maternal age, marital status, educational status, occupational status, monthly income, ethnicity, religion and support from relatives. Maternal health service and related characteristics such as place of delivery, mode of delivery, time when breastfeeding started, prelactile feeding, harmful cultural feeding practice and counseling service about feeding.

### Operational definition

#### Minimum dietary diversity (MDD)

The proportion of children 6–23 months of age who receive foods from 4 or more food groups with the food groups consisting; (I) grains, roots, and tubers; (II) legumes and nuts; (III) dairy products; (IV) flesh foods; (V) eggs; (VI) vitamin A-rich fruits and vegetables; and (vii) other fruits and vegetables during the previous day [7].

#### Minimum meal frequency (MMF)

Feeding of infant and young children (IYC) that fulfills at least 2–3 times complementary feeding within 24 h for IYC age of 6–8 month and 3–4 times complementary feeding within 24 h for IYC age of 8 months and above [7].

#### Minimum acceptable diet (MAD)

Is the combination of both minimum dietary diversity and meal frequency [8].

#### Harmful cultural feeding practices

Having practiced any one of the following is considered as harmful cultural feeding practices. Food taboos, avoiding colostrum, delay initiation of breastfeeding, giving butter and/or water for a newborn due to religious and cultural reasons [9].

#### Appropriate complementary feeding practice

This is quantified using a composite indicator comprising the WHO score and IYCF indicators that relate closely to complementary feeding [7].

### Data analysis

After data collection, the responses in the completed questionnaire were coded and entered into Epi data version 4.1 and were exported to Statistical Packages for Social Sciences (SPSS) version 25.0. It was cleaned and edited (check for missing values and outliers) accordingly. The outcome variable was coded as ‘1’ for appropriate complementary feeding practice and ‘0’ for inappropriate complementary feeding practice. Descriptive statistical analysis was performed to compute the frequency, percentage, and mean for continuous independent variables. Before the analysis, the assumptions of the chi-squared test were checked. When smaller expected frequencies were encountered, re-categorization of variables or merger of the levels was performed. Binary logistic regression was performed to determine the crude relationship of each independent variable with appropriate complementary feeding practices and to select candidate variables for multivariable logistic regression analysis. Variables with a *p*-value < 0.25 in the binary logistic regression analysis were entered into multivariable logistic regression. The assumptions of the logistic regression were also checked. Hosmer- Lemeshow tests for goodness of fit were carried out and found to be a good fit (*p* = 0.87(> 0.05)). Finally, the results were presented in texts, tables, graphs and discussed using the odds ratio and 95% confidence level.

### Statement of confirmation

All methods aforementioned above were carried out in accordance with relevant guidelines and regulations.

## Results

### Socio-demographic characteristics

A total of 257 study participants participated in the study, resulting in a response rate of 97.5%. Half (52.1%) of the infants and young children were female. Among the respondent mothers, 109(42.2%) were between the age group of 25–29. Almost all (96.5%) of the participants were Orthodox Christian followers. Of the mothers who participated in the study 158(61.5%) were illiterate, 181(70.4%) were housewives and 238(92.6%) were married. Most husbands’ occupations were farmers (49.8%) and the mean number of family members and under-five children in the study area was 4 (±1.7) and 1 (±0.6) respectively (Table 1).

**Table 1:** Socio-demographic characteristics of mothers/caregivers of 6–23 month children at Gendewuha, Amhara Region, Ethiopia 2020

Variable	Category	Frequency(n)	Percentage(%)
Sex of the child	Female	134	52.1
	Male	123	47.9
Age of child (in months)	6–11	58	31.1
	12–17	86	33.1
	18–23	113	35.8
Religion	Orthodox	248	96.5
	Other^a^	9	3.5
Ethnicity	Amhara	18	7.0
	Kmant	239	93.0
Age of the mother (in years)	18–24	57	22.2
	25–29	109	42.4
	30–34	57	22.2
	> 35	34	13.2
Education level of the mother	No formal education	158	61.5
	Primary	63	24.5
	Secondary and above	36	14.0
Occupation of the mother	Housewife	181	70.4
	Other^b^	76	29.6
Marital status	Single	14	5.4
	Married	238	92.6
	Other^c^	5	1.9
Monthly income	< 999	8	10.9
	1000–2999	107	41.6
	3000–3999	30	11.7
	> = 4000	92	35.8
Getting support from relatives	Yes	16	6.2
	No	241	93.8

Other^a^Muslim, Catholic Other^b^Employed, Private, daily laborer Other^c^Divorced, Widowed

### Maternal health care and child complementary feeding practice

The mean number of pregnancy and antenatal care (ANC) visits was 3(±1.6) and 3(±1.4) respectively. Regarding mode of delivery, almost all (93%) mothers delivered through spontaneous vaginal delivery, four-fifths (80.9%) gave birth at a health institution, and 45.1% of women had a birth interval of 2 years. About 85.2% of mothers started breastfeeding within 1 h of delivery and only 13.6% of women practiced prelactal feeding. Cow milk was the most commonly used type of complementary feeding (33.5%). Approximaly half (50.6%) of mothers reported that they practiced harmful cultural feeding practices and almost all mothers (93.4%) reported that their current feeding practices were not similar to those before they were displaced (Table 2).

**Table 2:** Maternal and child-related factors of mothers of children 6–23 months at Gendewuha, Amhara Region, Ethiopia 2020

Variable	Category	Frequency(n)	Percentage(%)
Place of delivery	Health Institution	208	80.9
	Home	49	19.1
Mode of delivery	Spontaneous vaginal delivery	239	93.0
	Cesarean section	18	7.0
Breastfeeding initiated	≤1 h	219	85.2
	> 1 h	38	14.8
Prelactile feeding	Yes	35	13.6
	No	222	86.4
Harmful Culture on complementary feeding	No	130	50.6
	Yes	127	49.4
Counseling about feeding	Yes	223	86.8
	No	34	13.2

### Dietary diversity

Only 14% of the mothers provided a diversified diet for their children aged 6–23 months. Among the seven food groups; grains, roots and tubers were commonly used, accounting for 89.9%. Only 32.7% of the mothers fed their children’s dairy products (Table 3).

**Table 3:** Types of complementary food introduced to children aged 6–23 months in Gendewuha, Amhara Region, Ethiopia 2020

Variable	Category	Frequency(n)	Percentage(%)
Grains, roots, tubers	Yes	231	89.9
	No	26	10.1
Vitamin A-rich fruits and vegetables	Yes	20	7.8
	No	237	92.2
Other fruits and vegetables	Yes	40	15.6
	No	217	84.4
Flesh foods	Yes	37	14.4
	No	220	85.6
Eggs	Yes	65	25.3
	No	192	74.7
Legumes	Yes	115	44.7
	No	142	55.3
Dairy products	Yes	84	32.7
	No	173	67.3

### Indicators for complementary feeding

**Fig. 1:**
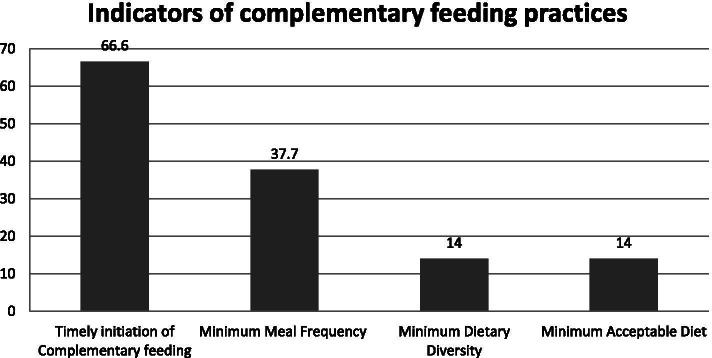
Indicators of complementary feeding of children aged 6-23 months (*n*=257) Gendewuha district, East Gondar zone, Ethiopia, 2020

The overall prevalence of appropriate complementary feeding practices was 26.8%. Indicators of complementary feeding were assessed and only 14% of mothers offered four or more food to their children which meeting the minimum dietary diversity criteria on the day preceding the survey. One-third (66.9%) of the mothers initiated complementary feeding at 6 months. About 37.7% of mothers met the minimum meal frequency according to child age category and their breastfeeding status on the day preceding the survey. The minimum acceptable diet for the children was 14% (Fig. 1).

### Factors associated with complementary feeding practice

In the binary logistic regression analysis, variables with a *p*-value < 0.25 were fitted into the multivariable logistic regression analysis. In the bivariable analysis variables such as child age, ethnicity, maternal age, presence of support from relatives, prelactal feeding and harmful cultural feeding practices were found to be significant factors of appropriate complementary feeding practices.

Finally, in the multivariable logistic-regression analysis all significant variables in binary logistic regression were controlled for possible confounding factors. The results showed that the mothers whose child age was between 6 and 11 months were 89% less likely (AOR = 0.11, 95%CI: 0.04, 0.27) to practice appropriate complementary feeding practice as compared to mothers whose child age was between 18 and 23 months. Similarly, mothers whose infant age was between 12 and 17 months were 65% less likely (AOR = 0.35, 95% CI = 0.17, 0.70) to practice appropriate complementary feeding than those mothers whose infants were between 18 and 23 months. The other predictor variable found to be significant factor was the harmful cultural child feeding practice. Those women who did not have harmful culture on complementary feeding were 2 times (AOR = 2.04; 95% CI: 1.06,3.96) more likely to practice appropriate complementary feeding as compared to their counterparts (Table 4).

**Table 4:** Binary and multivariable logistic regression analysis with appropriate complementary feeding practice among children aged 6–23 months at Gendewuha, Amhara Region, Ethiopia 2020

Variable	Category	Appropriate complementary feeding practice		COR (95% CI)	AOR (95% CI)
		Yes	No		
Child age (month)	6–11	7 (8.8%)	73 (91.2)	0.11 (0.05, 0.27)	0.11 (0.04,0.27) ^a^
	12–17	20 (23.5)	65 (76.5)	0.37 (0.19,0.70)	0.35 (0.17,0.70) ^a^
	18–23	42 (45.7)	50 (54.3)	1	1
Ethnicity	Amhara	2 (11.1)	16 (88.9)	0.32 (0.07,1.43)	0.43 (0.08,2.24)
	Kmant	67 (28)	172 (72)	1	1
Maternal age	18–24	9 (15.8)	48 (84.2)	0.27 (0.10,0.72)	0.65 (0.18,2.39)
	25–29	28 (25.7)	81 (74.3)	0.50 (0.22,1.12)	0.83 (0.29,2.38)
	30–34	18 (31.6)	39 (68.4)	0.66 (0.27,1.59)	1.07 (0.34,3.36)
	> 35	14 (41.2)	20 (58.8)	1	1
Support from relatives	Yes	1 (6.2)	15 (93.8)	0.17 (0.02,1.31)	0.35 (0.04,3.07)
	No	68 (28.2)	173 (71.8)	1	1
Prelactile feeding	Yes	5 (14.3)	30 (85.7)	0.41 (0.15,1.11)	0.49 (0.16,1.50)
	No	64 (28.8)	158 (71.2)	1	1
Harmful Cultural feeding practice	No	40 (30.8)	90 (69.2)	1.50 (0.86,2.62)	2.04 (1.06,3.96) ^a^
	Yes	29 (22.8)	98 (77.2)	1	1

*COR* Crude Odd Ratio, *AOR* Adjusted Odd Ratio

*p-value<0.05

## Discussion

The findings of the current study revealed that the level of appropriate complementary feeding was 26.8%. This finding is higher than studies from Tigray, Ethiopia (10.75%), ArsiNegele, Ethiopia (9.5%), and Northern Ghana (13.8%) [10–12]. However, this study is much lower than the studies conducted in Lasta district, Ethiopia (56.5%), Enemay district, Ethiopia (40.5%), Debre Tabor hospital, Ethiopia (37.2%) [13–15]. This could be due to the difference in the stability of the population between the host population and internally displaced populations. In addition, internally displaced mothers might not access and afford a variety of foods as they probably leave their homes in an emergency.

According to the WHO recommendation, complementary feeding should be timely and started at the age of 6 months. In the current study, approximately 66.6% of mothers/caregivers started complementary feeding at 6 months of age, which is consistent with studies at Benshangul and Debre Tabor hospital reported as 62–67% [15, 16]. However, it was detected to be higher than the national health survey report (60%), Lasta district, Ethiopia, (57.7%), and Kismayo, Somalia (49.2) [2, 3, 17]. Besides, this finding is lower than that of the study conducted in ArsiNegele, Ethiopia (72.5%), Tigray, Ethiopia (79.7%), Enemay district, Ethiopia (71.3%), Akpabuyo area, Nigeria (85.4%), Tanzania (92.3%), Indonesia (87.3%) and Kachin state (79%) [10, 11, 14, 18–21].

It is recommended that infants and young children consume foods from at least four different food groups in addition to breast milk. This study showed that the minimum dietary diversity score of children 6–23 months was about 14% which was similar to the EDHS report of 2016(14%) [2] and higher than a study conducted in Kismayo, Somalia (8.7%) and Northwest Ethiopia, Enemay district (8.5%) [14, 17]. In contrary, the finding of this study was lower than studies conducted in Lasta district, Ethiopia (60.7%), Benshangul, Ethiopia (23.7%), Ghana (35.6%), Nigeria (31.5%), Tanzania (38.2%), and a study in Ukraine (93.2%) [12, 13, 16, 19, 20, 22].

This study also depicted that the prevalence of MAD was 14%. This finding was higher than studies done in Tigray, Ethiopia (11.9%), EDHS (7%), Akpabuyo area, Nigeria (7.3%), and Kismayo, Somalia (3.3%) [2, 11, 17, 19]. However, this finding was lower than studies in Ghana (24.9%), Tanzania (15.9%), and Indonesia (44.9%) [12, 20, 21]. This might be due to the usual ignorance of the internally displaced populations for food and nutritional security compared to the country-to-country refugees by different humanitarian aid and non-governmental organizations. In addition to this, the types of foods given to IDPs in the study area lack animal source food and vitamin reach fruits and vegetables.

The findings of this study also revealed that child age and not having harmful culture on complementary feeding were predictors of complementary feeding practices. In this study, mothers of children aged 6–11 months were 89% times & 12–17 months were 65% times less likely to practice appropriate complementary feeding as compared to mothers of children aged 18–23 months. This finding is consistent with the previous studies conducted in Tigray, Ethiopia, ArsiNegele, Ethiopia, Enemay district, Ethiopia, Ghana, Tanzania, and Indonesia [10–12, 14, 20, 21]. This could be explained by children aged 6–11 and 12–17 months needing special attention for complementary feeding and tiresome feeding. Therefore, the mothers might prefer to feed breast milk instead of complementary food in this age category.

Mothers who did not have harmful culture on complementary feeding were 2 times more likely to practice appropriate complementary feeding practices as compared to their counterparts. This is supported by the study conducted in Kenya which outlines cultural beliefs and taboos (food taboos, restrictions, beliefs associated with certain foods) have a strong influence on infant feeding and undermine optimal infant feeding practices; breastfeeding and complementary feeding [23]. Furthermore, the type of foods consumed and the feeding habits in the community where they were displaced might negatively affect the complementary feeding practice of the IDP mothers.

Even though this study addressed complementary feeding practice in the study area, the study is not free from some limitations. Information about several associated factors of complementary feeding practice previously reported, such as knowledge, attitude and practices toward complementary feeding practice and other pertinent variables (like household condition) are missed. Seasonal variations might affect the food groups consumed by the infants and young children during the time of the interview. The study design itself poses difficulty in establishing a cause-effect relationship and there might be the possibility of recall bias because mothers/caregivers were asked to report events that occurred months or sometimes ago.

## Conclusion

The proportion of mothers who practiced timely complementary feeding, follow acceptable meal frequency, dietary diversity, minimum acceptable diet, and the overall ACFP were found to be low. Child age and having harmful culture on complementary feeding were found to be the two predictors of the complementary feeding practice of IDP mothers in the study area.

## Supplementary Information

**Additional file 1.**

## Data Availability

Full data set and other materials on this study can be obtained from the corresponding author on reasonable request.

## Abbreviations

APHI: Amhara Public Health Institute
ARDPFSCO: Amhara Region Disaster Prevention and Food Security Coordination Office
EBF: Exclusive Breast Feeding
EDHS: Ethiopian Demographic and Health Survey
ENA: Essential Nutrition Action
IDP: Internally Displaced People
IYCF: Infant and Young Child Feeding
MAD: Minimum Acceptable Diet
MDD: Minimum Dietary Diversity
MMF: Minimum Meal Frequency
NBCC: Nutrition Behavioral Change Communication
NDRMC: National Disaster and Risk Management Commission
NGO: Non-Governmental Organization
NNP: National Nutrition Program
UNICEF: United Nations International Children’s Emergency Fund
WHO: World Health Organization

## References

[1] Organization WH (2003). Global Strategy for Infant and Young Child Feeding.

[2] Ethiopia demographic and health survey 2016: key indicators report. The DHS Program ICF. 2016.

[3] Organization UN (2001). Guiding principles on internal displacement.

[4] White JM, Bégin F, Kumapley R, Murray C, Krasevec J (2017). Complementary feeding practices: Current global and regional estimates. Matern Child Nutr.

[5] Mahalingam S, Narayan G, van der Velde E (2002). The rights of internally displaced children: selected field Practices from UNICEF’s experience. Refuge: Can J Refugees.

[6] Poeter G, Olivier G (2017). Assessment of complementary feeding practice among internally displaced mother-child pair in south Sudan.

[7] World Health Organization (2010). Indicators for assessing infant and young child feeding practices part 2: measurement.

[8] Jones AD, Ickes SB, Smith LE, Mbuya MN, Chasekwa B, Heidkamp RA, Menon P, Zongrone, AA, Stoltzfus RJ. World Health Organization infant and young child feeding indicators and their associations with child anthropometry: a synthesis of recent findings. Matern Child Nutr. 2014;10(1):1–17.10.1111/mcn.12070. 10.1111/mcn.12070 PMC6860255 23945347

[9] Abebe H, Beyene GA, Mulat BS (2021). Harmful cultural practices during perinatal period and associated factors among women of childbearing age in southern Ethiopia: community based cross-sectional study. PLoS One.

[10] Kassa T, Meshesha B, Haji Y, Ebrahim J (2016). Appropriate complementary feeding practices and associated factors among mothers of children age 6–23 months in southern Ethiopia, 2015. BMC Pediatr.

[11] Mekbib E, Shumey A, Ferede S, Haile F (2014). Magnitude and factors associated with appropriate complementary feeding among mothers having children 6–23 months-of-age in northern Ethiopia; a community-based cross-sectional study. J Food Nutr Sci.

[12] Saaka M, Larbi A, Mutaru S, Hoeschle-Zeledon I (2016). Magnitude and factors associated with appropriate complementary feeding among children 6–23 months in northern Ghana. BMC Nutrition.

[13] Molla M, Ejigu T, Nega G (2017). Complementary Feeding Practice and Associated Factors among Mothers Having Children 6–23 Months of Age, Lasta District, Amhara Region, Northeast Ethiopia. Adv Public Health.

[14] Gessese D, Bolka H, Alemu Abajobir A, Tegabu D (2014). The practice of complementary feeding and associated factors among mothers of children 6-23 months of age in Enemay district, Northwest Ethiopia. Nutr Food Sci.

[15] Dagne AH, Anteneh KT, Badi MB, Adhanu HH, Ahunie MA, Aynalem GL (2019). Appropriate complementary feeding practice and associated factors among mothers having children aged 6–24 months in Debre Tabor hospital, north West Ethiopia, 2016. BMC Res Notes.

[16] Ayana D, Tariku A, Feleke A, Woldie H (2017). Complementary feeding practices among children in Benishangul Gumuz region, Ethiopia. BMC Res Notes.

[17] Infant and young children feeding knowledge, attitude and practice survey report among Kismayo IDP's, Somalia.2016:55.

[18] ProPAN Assessment of Infant and Young Child Feeding Practices in IDP Camps in Kachin State. 2015 144.

[19] Udoh EE, Amodu OK (2016). Complementary feeding practices among mothers and nutritional status of infants in Akpabuyo area, Cross River State Nigeria. Springerplus.

[20] Victor R, Baines SK, Agho KE, Dibley MJ (2014). Factors associated with inappropriate complementary feeding practices among children aged 6–23 months in T Tanzania. Matern Child Nutr.

[21] Ng CS, Dibley MJ, Agho KE (2012). Complementary feeding indicators and determinants of poor feeding practices in Indonesia: a secondary analysis of 2007 demographic and health survey data. Public Health Nutr.

[22] Summers A, Bilukha OO (2018). Suboptimal infant and young child feeding practices among internally displaced persons during conflict in eastern Ukraine. Public Health Nutr.

[23] Karigi LN, Mutuli LA, Bukhala P (2016). Socio-cultural practices and beliefs influencing infant and young child feeding in lubao sub-location Kakamega Country. J Nutr Health Food Eng.

