# Supplementary material for: Complementary feeding practice and associated factors among internally displaced mothers of children aged 6–23 months in Amhara region, Northwest Ethiopia: a cross-sectional study
Source: BMC Pediatr. 2021 Dec 20;21:583. doi: 10.1186/s12887-021-03050-y (PMC8686606; doi:10.1186/s12887-021-03050-y)
Supplement: Supplementary file 1 — Additional file 1. [file 12887_2021_3050_MOESM1_ESM.docx]

## English and Amharic version Questionnaire

## Annex I: Participant information sheet, English version

**How to approach and interview the mother/care givers (for data enumerator use only)**

- Introduce yourself by name to mother/ care giver and explain the purpose of your visit survey explicitly according to the participant information sheet. Informing all the participant information sheet content is mandatory.
- Take informed consent agreement if the mother/care giver has already agreed before starting interview.
- Conduct interview for each mother/care giver separately (in private)
- At the end of the interview, tell the mother/care giver that it is the end of the interview and say thank you very much for giving her time.

## Annex II: Subject information sheet

Good morning/afternoon. My name is _____and I am from ____. I am a member of a data collector team on behalf of the study conducted by Meron Zebene et al who is a Masters of public health nutrition in St. Paul Hospital Millennium Medical College.

**Title of the project**: complementary feeding practice and its associated factors among internally displaced mothers of children aged 6-23 months Gendewuha district, west Gondar, Ethiopia

**Investigators:** Meron Zebene et al

**Coordinating office**: Saint Paul Hospital Millennium Medical College

**Introduction**: Appropriate complementary feeding practice is low in most parts of sub-Saharan countries, including Ethiopia. Gendewuha is a district where IDP’s are collectively located. So this study assess the level of appropriate complementary feeding practice and its associated factors among internally displaced mothers of children aged 6-23 months in Gendewuha district.

**Purpose**: The purpose of this research is to determine level of appropriate complementary feeding practice and its associated factors among internally displaced mothers of children aged 6-23 months in Gendewuha district, west Gondar, Ethiopia. This research is undertaken for partial fulfillment of master’s thesis in Public health nutrition.

**Procedure and participation**: The method of this research is a cross-sectional study design. The expected duration of the study participant with the interviewer will not be more than 25 minutes. You will be asked to participate in this research since the truthful information which you will provide is important for the understanding of the proposed research. However, your particular participation is affirmed by the procedure of probability sampling technique which provides equal chance of selection.

**Confidentiality**: In order to establish secured safeguards of the confidentiality of research data, the data collector will use codes during the data collection period instead of using names. The original data will be locked in cabinets until the data analysis is carried out and no person shall have access to it except the principal investigator and the supervisor for data checking and cleaning purposes. The use of information for any purpose other than that to which participants consent is unethical to the participants. The information you provide is not disclosed in the way that may identify your personal characteristics or violate privacy.

**Benefit**: The research does not have short term financial, healthcare and capacity-building benefits to the research participant as an individual or as a group. In the long run, the outcome of this study will help the concerned organizations and policy makers in consideration, direction and formulation of strategy and design of programs for IDP’s.

**Risk**: The research does not have any inhuman treatment of the research participants or any physical harm, social discrimination, psychological trauma and economic loss.

**Inducement, incentive and compensation**: This study process will not have any form of inducement, or coercion and the study does not bring any risks that incur compensation.

**Freedom to withdraw**: If you want to participate in the study, you have also the full right to withdraw from the study at any time you wish without any penalty. Nobody will ask or enforce you to explain the reason for withdrawal.

**Person to contact**: You have the right to ask information that is not clear about the research context and content before and during the research work. You can contact the principal investigator and the data collector’s supervisor. In addition this research had undergone ethical review and approval by Saint Paul Hospital Millennium Medical College, college of public health. If you want more information about this research project, you can contact the following people.

1. Saint Paul Hospital Millennium Medical College, School of public health

2. Investigator name and address: Meron Zebene et al Telephone:+251-966728710

## Annex III: Informed consent agreement form, English version

**Title of the research:** complementary feeding practice and its associated factors among internally displaced mothers of children aged 6-23 months in Gendewuha district, west Gondar, Ethiopia. I am aware that this research undertaking conducted by investigator Meron Zebene et al. I have been also fully informed in the language I understood and about the research project objective to assess complementary feeding practice and its associated factors among internally displaced mothers of children aged 6-23 months in Gendewuha district, west Gondar, Ethiopia. I have been informed that all the information I shall provide to the interviewer will be kept confidential. I understood that the research has no any risk and no compensation. I also know that I have the right to withhold information, skip questions to answer or to withdraw from the study any time. I have been informed that nobody will impose on me to explain the reason of withdrawal. It is also clear that there will be no effect at all in my health benefit or other administrative effect that I get from the district. I have been assured of the right to ask information that is not clear about the research before and/or during the research work.

I have read this form (it has been read to me in the language I comprehend) and I understood the condition stated above; therefore, I am willing and confirm my participation by signing this consent form. Mothers/caregivers agreed to participate in the study: (Mark one of them for verbal/oral consent)

Yes __________

No __________

Name of interviewer ________________

Signature __________________ Date ____________________

Thank you for your participation

## Annex IV. English version Questionnaires

How are you?

My name is ………………….…... I am collecting data for the study done by SPHMMC on magnitude and associated factors of complementary feeding practice among 6-23 month IDP children in Gendewuha. I am going to ask you some questions that will be relevant to this study. Any information collected will be treated with confidentiality.

Date of assessment……/……/……

Time of assessment begin …….ends……………………Code…………………..

| **SECTION ONE- SOCIO DEMOGRAPHIC CHARACTERISTICS** | | | | | |
| --- | --- | --- | --- | --- | --- |
| 101 | Sex of the child | 1. Female 2. Male |  |  | |
| 102 | Birthdate | Day/Month/Year…..\|___\|___\| /\|___\|___\| / \|___\|___\|\| |  |  | |
| 103 | Child’s age in completed months | ………………………………. |  |  | |
| 104 | Age of the mother/care giver in year | Age in completed year ---------------- |  |  | |
| 105 | Ethnicity | 1. Amahra 2. Kmant 3. Other specify_____ |  |  | |
| 106 | Where you displaced | ______________________ |  |  | |
| 107 | Religion | 1. Orthodox 2. Muslim 3. Catholic 4. Protestant 5. Others specify_______ |  |  | |
| 108 | Educational status of the mother/care giver | 1. No formal education 2. Primary education 3. Secondary 4. College and above |  |  | |
| 109 | Occupation status | 1. House wife 2. Daily laborer 3. Government / private / employee 4. Private business 5. Others specify __________ |  |  | |
| 110 | Marital status | 1. Single 2. Married 3. Divorce 4. Widowed |  |  | |
| 111 | Educational status of the partner | 1. No formal education 2. Primary education 3. Secondary 4. College and above |  |  | |
| 112 | Occupation status of the partner | 1. Daily laborer 2. Government / private / employee 3. Private business 4. Farmer 5. Other specify________ |  |  | |
| 113 | Family size | ------------- |  |  | |
| 114 | Number of under five children in the household | -------------- |  |  | |
| 115 | Monthly income of the household | -----------------------------birr |  |  | |
| 116 | Do you have support from relatives? | 1. Yes 2. No |  |  | |
| **SECTION TWO: Maternal related factors** | | | | | |
| 201 | The Number of antenatal care visit attended in the index pregnancy | .………….. |  |  | |
| 202 | Number of pregnancy | ……………… |  |  | |
| 203 | If two and above, what was the birth interval? | .………….. |  |  | |
| 204 | Place of Delivery | 1. Health institution 2. Home |  |  | |
| **SECTION THREE- Feeding practices and related factors** | | | | | |
| 301 | Does the baby ever breast fed | 1. Yes 2. No | If yes skip to 303 | |  |
| 302 | What type of feeding option do you use for your child? | 1. Infant formula feeding 2. Cow milk |  | |  |
| 303 | When does the breast feeding started | 1. Within one hour 2. After one hour |  | |  |
| 304 | Any food given before the first breast feeding after birth | 1. Yes 2. No |  | |  |
| 305 | Age in months complementary foods introduced | …………………. |  | |  |
| 306 | Type of complementary food introduced | 1. Cow milk 2. Commercial infant formula 3. Porridge/maize, wheat, Sorghum, teff 4. Fortified porridge (porridge with milk, fat, egg, added 5. Semisolids (e.g. mashed potatoes, bananas beans, etc 6. Water and sugar 7. Others (Specify)………… |  | |  |
| 307 | Reason to introduce additional foods | 1. Baby crying after feeding 2. It was correct time 3. Suckling to much 4. Clinician’s advice 5. Lack of enough breast milk 6. Hard to report to work 7. Infant refusal to BF 8. Fear of HIV transmission 9. Others (specify)……………………. |  | |  |
| 308 | Are you still breastfeeding? | 1. Yes 2. No | If No skip to 310 | |  |
| 309 | If Yes to Q308 when is your plan to stop? | Specify the age……………… |  | |  |
| 310 | Why are planning to stop at the age specified on Q309? | Mention the reason…………… |  | |  |
| 311 | If no to Q308, when did you stop breastfeeding? | …………………. |  | |  |
| 312 | If no to Q308, why did you stop breastfeeding? | 1. Infant illness 2. Maternal illness 3. Fear of vertical transmission 4. Others specify………………. |  | |  |
| 313 | Counseling about feeding | 1. Yes 2. No |  | |  |
| 314 | How many times do you feed your child over the past 24 hrs? | Specify……… |  | |  |
| 315 | Does your child encounter any illness in the preceding two weeks? | 1.Yes  No |  | |  |
| 316 | Does the culture affect your feeding practice? (do you practiced any one of the following? Food taboos, avoiding colostrum, delay initiation of breastfeeding, giving butter and/or water for a newborn) | 1. Yes 2. No |  | |  |
| 317 | Is your current feeding practice similar with the previous one, before you were displaced? | 1. Yes 2. No |  | |  |

**Instruction:** Read listed food examples and then circle *‘one’* in the box if the child ate the food in question or circle ‘*two’* in the box if the child didn’t ate any of the food lists in a food group.

| **SECTION Four :Dietary Diversity Questionnaire For Children** | | | |
| --- | --- | --- | --- |
| Now I would like to ask you about the type of foods that your child ***ate yesterday during the day and at night***. Please describe the foods (meals and snacks) that your child ate yesterday during the day and night, whether at home or outside the home. Start with the first food eaten in the morning. | | | |
| **Code** | **Food groups** | **Examples** | **Response** |
| **401** | Grains, roots and tubers | Bread, noodles, biscuits, cookies or any other foods made from millet, sorghum, maize, rice, wheat or white potatoes, white yams, cassava, or foods made from roots or sweet potatoes that are yellow or orange inside. | 1.Yes  2.No |
| **402** | Vitamin A-rich plant foods | Ripe mangoes, papayas + other locally available vitamin A-rich fruits or Pumpkin, carrots, squash. | 1.Yes  2.No |
| **403** | Other fruits and vegetables | Other fruits including wild fruits or Any dark, green, leafy vegetables such as cassava leaves, bean leaves kale, spinach, pepper leaves, or other vegetables. | 1.Yes  2.No |
| **404** | Meat, poultry, fish and sea food | Beef, goat, chicken, Liver, kidney, heart or other organ meats or blood-based foods or Any fresh or dried fish or shellfish. | 1.Yes  2.No |
| **405** | Eggs | Egg | 1.Yes  2. No |
| **406** | Legumes, pulses and nuts | Beans, peas, lentils, nuts, seeds or foods made from these. | 1.Yes  2. No |
| **407** | Dairy products | Any cheese, yogurt, milk or other milk products. | 1.Yes  2. No |

Source: Partially adapted from the following sources

1. UNICEF/WHO. Indicators for assessing infant and young child feeding practices. Part 1. Definitions. Geneva: World Health Organization, 2000
2. UNICEF/WHO. Indicators for assessing infant and young child feeding practices. Part 2. Measurement. Geneva: World Health Organization, 2010
3. Ethiopian Public Health Institute. Ethiopian Demographic and Health Survey, infant and young child feeding practice questionnaires.2016
4. FANTA infant and young child DD tool

**Amharic version Questionnaire**

**አባሪ**

**አባሪ 1፡የመረጃሰብሳቢዎችየመረጃቅፅ፤አማረኛፎርም**

(መረጃ ሰብሳቢዎች እናቶችን እንዴት ቃለመጠይቅ እንደሚያደርጉ መመሪያ፤ለመረጃ ሰብሳቢዎች ብቻ የሚያገለግል)

- እራስዎን በማስተዋዎቅ ይጀምሩ፤የጥናቱን ርዕስ፣አላማ በዝርዝር ለእናቶች ይግለዑ
- ቃለ-መጠይቁን ከመጀመረዎ በፊት የወላጅ ስምምነት ይዉሰዱ
- እያንዳንዱን እናቶችን ቃለ-መጠይቁን ለብቻቸዉ ይጠይቁ
- ቃለ-መጠይቁ ሲጠናቀቅ፣ እንደተጠናቀቀ ለእናቶች ይንገሩ፣ መረጃውን በመስጠት ስለተባበሩም እናቶችን ያመስግኑ፡፡

**አባሪ 2፡የተሳታፊዎችየመረጃቅፅ**

እንደምን አደሩ/ዋሉ፡፡ ስሜ-----------እባላለሁ፡፡ የማስተርስ ዲግሪን በቅዱስ ጳዉሎስ ሆስፒታል ሚሊኒየም ሜዲካል ኮሌጅ በከፊል ለማሟላት በሜሮን ዘበነ አማካኝነት በሚደረገው ጥናት ውስጥ በመረጃ ሰብሳቢነት አገለግላለሁ፡፡

**የጥናቱርዕስ፡**በገንደዉሃ አካባቢ ተፈናቅለዉ በሚኖሩከ 6-23 ወር የሚሆናቸዉ ህጻናት እናቶች ትክክለኛ የሆነ ተጨማሪ ምግብ ልጆቻቸዉን እየመገቡ መሆናቸዉን እና በቂ ተጨማሪ ምግብ እንዳይመገቡ የሚያደርጉ ተጓዳኝ ምክንያቶች

ዋና ተመራማሪ ፡ሜሮንዘበነ

የጥናቱተቆጣጣሪ፡ ኤርሚያስ

**የጥናቱመሪ፡**ቅዱስ ጳዉሎስ ሆስፒታል ሚሊኒየም ሜዲካል ኮሌጅ የህብረተሰብ ጤናት/ቤት

**መግቢያ፡**ትክክለኛ የሆነ ተጨማሪ ምግብ አመጋገብ ኢትዮጵያን ጨምሮ ከሰሃራ በታች ያሉ አፍሪካ ሀገራት ላይ አነስተኛ ነዉ፡፡ ገንደዉሃ ተፈናቅለዉ የሚኖሩ የህብረተሰብ ክፍሎች ተሰባስበዉ የሚገኙበት አካባቢ ሲሆን ይህ ጥናት በዚህ የሚኖሩ ከ 6-23 ወር የሚሆናቸዉ ህጻናት እናቶች ትክክለኛ የሆነ ተጨማሪ ምግብ ልጆቻቸዉን እየመገቡ መሆናቸዉን እና በቂ ተጨማሪ ምግብ እንዳይመገቡ የሚያደርጉ ተጓዳኝ ምክንያቶችን የሚለይ ይሆናል፡፡

**የጥናቱአላማ፡**የዚህ ጥናት አላማ በገንደዉሃ አካባቢ ተፈናቅለዉ በሚኖሩ ከ6-23 ወር የሚሆናቸዉ ህጻናት እናቶች ትክክለኛ የሆነ ተጨማሪ ምግብ ልጆቻቸዉን እየመገቡ መሆናቸዉን እና በቂ ተጨማሪ ምግብ እንዳይመገቡ የሚያደርጉ ተጓዳኝ ምክንያቶችን መለየት ነዉ፡፡ይህ ጥናት የሚደረገው የማስተርስ ዲግሪን በቅዱስ ጳዉሎስ ሆስፒታል ሚሊኒየም ሜዲካል ኮሌጅ በከፊል ለማሟላት ነው፡፡

**የጥናቱቅደምተከተልናየተሳትፎሁኔታ፡**ጥናቱ በአንድ ወቅት ተሰብስቦ የሚያልቅ ነው፡፡ቃለ-መጠይቁን ለማካሄድ የሚፈጀው ሰዓት 25 ደቂቃ ብቻ ነው፡፡በቃለ-መጠይቁ እንዲሳተፉ የምንጠይቅዎት መረጃው ጥናቱን ለማካሄድ በጣም አስፈላጊ ሆኖ ስላገኘነው ነው፡፡እርስዎ የተመረጡትም ከሁሉም ሰው ጋር እኩል እጣ ውስጥ ከገቡና እጣው ከወጣልዎት በሁዋላ ነው፡፡

**ምስጢራዊነት፡**የሚሰጡት መረጃ ምስጢራዊነቱ እንዲጠበቅ የቻልነዉን ሁሉ እናደርጋለን፡፡ መረጃውን ስንሰበስብ የእርስዎን ወይም የልጅዎን ስም አንገልፅም፡፡ በዚህ ፋንታ የመለያ ቁጥሮችን እንጠቀማለን፡፡ የተሰበሰበው መረጃም የጥናት ትንተና እስኪሰራ ድረስ ከዋናው ተመራማሪና ከጥናቱ ተቆጣጣሪ በስተቀር ማንም በማያገኘው ሁኔታ ተቆልፎ ይቀመጣል፡፡ መረጃውን ስምምነት ከተፈፀመለት ዓላማ ውጭ መጠቀም ህገ ወጥ ስራ ነው፡፡

**በጥናቱ መሳተፍ ሊያስገኝ የሚችለው ጥቅም፡** የጥናቱ ተሳታፊዎች የሚያገኙት የአጭር ጊዜ የገንዘብም ሆነ የስልጠና ጥቅም አይኖርም፡፡ ነገር ግን በረጅም ጊዜ ሂደት የሚመለከታቸው አካላት የጥናቱን ግኝት መሰረት አድርገው ተፈናቅለዉ ለሚኖሩ የህብረተሰብ ክፍሎች የሚጠቅሙ ፕሮግራሞችን ሊነድፉ ይችላሉ፡፡

**በጥናቱመሳተፍሊያመጣየሚችለውጉዳት፡**ጥናቱሲካሄድኢ-ሰብዓዊየተሳታፊዎችአያያዝአይኖረም፡፡በጥናቱወቅትምንምአይነትአካላዊ፣ማህበራዊመገለል፣የስነ-ልቦናጉዳትእናየኢኮኖሚክስረትአያጋጥምም፡፡

**ማበረታቻወይምማካካሻክፍያበተመለከተ፡**ይህጥናትምንምአይነትተሳታፊዎችንአደጋላይየሚጥልሁኔታየለዉም፡፡ምንምአይነትየማበረታቻምይሁንየማካካሻክፍያአይኖረውም፡፡

**ጥናቱንየማnረጥነፃነት፡**በጥናቱለመሳተፍከተስማሙበማንኛውምሰዓትጥናቱንየማቋረጥመብትአለዎት፡፡በዚህምምክንያትየሚደርስብዎትምንምአይነትቅጣትአይኖርም፡፡ማንምአካልያቋረጡበትንምክንያትእንዲገልፁሊያሥገድድዎትአይችልም፡፡

**ተጨማሪመረጃሲያስፈልግየሚያናግሩት**አካል፡ከጥናቱበፊትምይሁንከጥናቱበኃላስለጥናቱግልፅያልሆነነገርካለተጨማሪመረጃመጠየቅይችላሉ፡፡ግልፅያልሆነልዎትንማንኛዉምጥያቄየጥናቱንዋናተመራማሪወይምየጥናቱንተቆጣጣሪበሚከተሉትአድራሻዎችአማካኝነትመጠየቅይችላሉ፡፡

1. ቅ/ጳዉሎስሆስፒታልሚሊንየምሜዲካልኮሌጅየህብረተሰብጤናት/ትክፍል
2. የጥናቱዋናተመራማሪስምንአድራሻ፡ሜሮንዘበነ፡ +251-966728710
3. የጥናቱተቆጣጣሪስምናአድራሻ፡ሳምሶን፡ +251-9

**አባሪ 3፡የወላጅእናቶችየስምምነትቅፅ፣አማረኛ**

**የጥናቱርዕስ፡**በገንደዉሃአካባቢተፈናቅለዉበሚኖሩከ 6-23 ወርየሚሆናቸዉህጻናትእናቶችትክክለኛየሆነተጨማሪምግብልጆቻቸዉንእየመገቡመሆናቸዉንእናበቂተጨማሪምግብእንዳይመገቡየሚያደርጉተጓዳኝምክንያቶችላይየሚደረግጥናትነው፡፡ይህጥናትበዋናተመራማሪሜሮን ዘበነለማተርስዲግሪማሟያየሚደረግጥናትመሆኑንተገንዝቢያለሁ፡፡የጥናቱዓላማበገንደዉሃአካባቢተፈናቅለዉበሚኖሩከ 6-23 ወርየሚሆናቸዉህጻናትእናቶችትክክለኛየሆነተጨማሪምግብልጆቻቸዉንእየመገቡመሆናቸዉንእናበቂተጨማሪምግብእንዳይመገቡየሚያደርጉተጓዳኝምክንያቶችንመለየት መሆኑንእኔበምረዳውቋንቋተገልፆልኛል፡፡ከዚህምበተጨማሪየምሰጠውመረጃምስጢራዊነቱየተጠበቀእንደሚሆንናበማንኛውምሰዓትጥናቱንማቋረጥብፈልግማቋረጥእንደምችልለዚህምምንምአይነትማብራሪያእንደማልጠየቅናበዚህምምክንያትምንምአይነትየማጣውየጤናምይሁንአስተዳድራዊጥቅምአለመኖሩንተገልፆልኛል፡፡ጥናቱበእኔላይየሚያመጣውጉዳትአለመኖሩንናማካካሻክፍያምአለመኖሩንተረድቻለሁ፡፡ከጥናቱበፊትምሆነበጥናቱወቅትግልፅያልሆነልኝንጥያቄመጠየቅእንድችልምየሚከተሉትአድራሻውችተሰጥተውኛል፡፡

1. ቅ/ጳዉሎስሆስፒታልሚሊንየምሜዲካልኮሌጅየህብረተሰብጤናት/ትክፍል
2. የጥናቱዋናተመራማሪስምንአድራሻ፡ሜሮንዘበነ፡ +251-966728710
3. የጥናቱተቆጣጣሪስምናአድራሻ፡ሳምሶን፡ +251-9

ይህንየስምምነትቅፅአንብቤዋለሁ (እኔበምረዳውቋንቋተነቦልኛል)፡፡ከላይየተገለፀውንተረድቼበጥናቱለመሳተፍየተስማማሁመሆኑንበፊርማዬአረጋግጣለሁ፡፡

እስማማለሁ---------------------------------

አልስማማም--------------------------------

የመረጃሰብሳቢውስም---------------------- ፊርማ--------------- ቀን------------

ስለተሳትፎዎእናመሰግናለን!!!

**አባሪ 4፡**አማረኛመጠይቅ

እንደምንአሉ?

ስሜይባላል፡፡ትክክለኛየሆነተጨማሪምግብልጆቻቸዉንእየመገቡመሆናቸዉንእናበቂተጨማሪምግብእንዳይመገቡየሚያደርጉተጓዳኝምክንያቶችበተሰኘርዕስየማስተርስዲግሪንበከፊልለማሟላትበገንደዉሃአካባቢተፈናቅለዉበሚኖሩከ6-23 ወርየሚሆናቸዉህጻናትእናቶችላይለሚሰራውጥናትመረጃሰብሳቢነኝ፡፡ለጥናቱአስፈላጊየሆኑጥያቄዎችንእጠይቅዎታለሁ፡፡ከእርስዎየምንወስደውመረጃሚስጥራዊነቱየተጠበቀነው፡፡

መጠይቁየተካሄደነትቀን--------/---------/----------------------

መጠይቁየተጀመረበትሰዓት----------------------መጠይቁየተጠናቀቀበትሰዓት----------------------- ኮድ ----------------------------

| ክፍልአንድ፡አጠቃላይየማህበረሰባዊእናእኮኖሚያዊሁኔታ | | | | | | | | | |
| --- | --- | --- | --- | --- | --- | --- | --- | --- | --- |
| የጥያቄተ.ቁ | | ጥያቄዎች | መልስ | | የሚዘለሉጥያቄዎች | | | ምርመራ | |
| 101 | | የህፃኑፆታ | ሴት…………………….1  ወንድ…………………..2 | |  | | |  | |
| 102 | | ህፃኑየተወለደበትወቅት | ቀን/ወር/ዓ.ም \|__\|___\|/\|___\|___\|/\|___\|___\|\|___\|___\| | |  | | |  | |
| 103 | | የህፃኑእድሜበወራት | \|__\|___\| ወር | |  | | |  | |
| 104 | | የእናት/የአሳዳጊእድሜበአመት | --------------------- | |  | | |  | |
| 105 | | ብሄር | ---------------------- | |  | | |  | |
| 106 | | የቀድሞ ቋሚ የመኖሪያ ስፍራ | ----------------------------- | |  | | |  | |
| 107 | | ሀይማኖት | ኦርቶዶክስ-----------------1  ሙስሊም-----------------2  ካቶሊክ-------------------3  ፕሮቴስታንት---------------4  ሌላካለይጥቀሱ-----------5 | |  | | |  | |
| 108 | | የእናት/የአሳዳጊየትምህርትሁኔታ | ያልተማረ---------------------1  ማንበብናመፃፍየሚችል------2  አንደኛደረጃ ---------------3  ሁለተኛደረጃ---------------4  ዲፕሎማናከዚያበላይ--------5 | |  | | |  | |
| 109 | | የእናት/የአሳዳጊየስራሁኔታ | የቤትእመቤት---------------1  የቀንሰራተኛ----------------2  የመንግስት/የግልተቀጣሪ-------3  የግል----------------------4  ሌላካለይጥቀሱ------------5 | |  | | |  | |
| 110 | | የእናት/የአሳዳጊ  የጋብቻሁኔታ | ያላገባ/ች-------------------1  ያገባ/ች--------------------2  የፈታ/ች-------------------3  የሞተነት/ባት---------------4  ሌላካለይጥቀሱ------------5 | |  | | |  | |
| 111 | | የባለቤትዎየትምህርትሁኔታ | ማንበብእናመጻፍየማይች---1  አንደኛደረጃ---------------2  ሁለተኛደረጃእናበላይ------3 | |  | | |  | |
| 112 | | የባለቤትዎየስራሁኔታ | የቀንሰራተኛ---------------1  የመንግስት/የግልተቀጣሪ-----2  የግል---------------------3  ሌላካለይጥቀሱ-----------4 | |  | | |  | |
| 113 | | የቤተሰብመጠን | ---------------------------- | |  | | |  | |
| 114 | | በቤት ዉስጥ ያሉ ከ5ዓመት በታች ህጻናት ብዛት | ----------------------------- | |  | | |  | |
| 115 | | የወር ገቢ( ካለ) | --------------------------- ብር | |  | | |  | |
| 116 | | ከሌላ ቤተሰብ ድጋፍ አሎት? | አዎ---------------1  አይ---------------2 | |  | | |  | |
| **ክፍልሁለት፡የእናቶችጤንናበተመለከተ** | | | | | | | | | |
| 201 | ይህንንህጻንባረገዝሽነትወቅትስንትጊዜለክትትልወደጤናተቋምሄደሻል? | | ---------------- | |  |  | | | |
| 202 | ስንትጊዜአርግዘሽታዉቂያለሽ? | | ------------------- | |  |  | | | |
| 203 | ሁለት እና ከዚያ በላይ ከሆነ የስንት አመት ልዩነት አለ? | | ----------------------------- | |  |  | | | |
| 204 | የትወለድሽ? | | ጤናተቋም---------1  ቤት--------------2 | |  |  | | | |
| 205 | በምን መንገድ ወለድሽ? | | በምጥ------------1  በቀዶጥገና---------2 | |  |  | | | |
| **ክፍላሶስት፡የአመጋገብሁኔታ** | | | | | | | | | |
| 301 | ህጻኑየእናትጡትጠብቷል? | | | አዎ---------1  አይደለም-----2 | | |  | |  |
| 302 | ህጻኑንለመመገብምንአይነትየአመጋገብአማራጭይጠቀማሉ? | | | የቆርቆሮወተት---------1  የላምወተት-----------2 | | |  | |  |
| 303 | ህጻኑጡትመጥባትመቼጀመረ? | | | በአንድ ሰዓት ዉስጥ---------1  ከአንድ ሰዓት በኋላ---------2 | | |  | |  |
| 304 | ህጻኑ ከተወለደ በኋላ ጡት ከመዉሰዱ በፊት የወሰደዉ ምግብ/ መጠጥ ነበር? | | | አዎ---------1  አይደለም-----2 | | |  | |  |
| 305 | ህጻኑ ስንት ወር ሲሆነዉ ተጨማሪ ምግብ ጀመረ? | | | -------------------------- | | |  | |  |
| 306 | የተጀመረዉተጨማሪምግብአይነትምንነበር? | | | ወተት(ከጡትወተትዉጪ)---------1  የቆርቆሮወተት---------2  ገንፎከበቆሎ/ስንዴ/ገብስ/ጤፍየተሰራ-------3  ምጥንገንፎ/ገንፎ፤ወተት/ቅባ/እንቁላልየተጨመረበት-------4  ጠጣርያልሆነምግብ-------5  ዉሃበስኳር-------6  ሾርባ-------7  ሌላካለይጥቀሱ-------8 | | |  | |  |
| 307 | ተጨማሪምግብአይነትየጀመሩነትምክንያትምንነበር? | | | የህጻኑከተመገበበኋላማልቀስ-------1  ትክክለኛዉሰዐትስለነበር-------2  ብዙስለሚጠባ-------3  በጤናባለሙያምክር-------4  በቂየጡትወተትባለመኖሩ-------5  የስራፍቃድስለማላገኝ-------.6  ህጻኑጡትአልጠባምስላለ-------7  ኤችአይቪቫይረስአንዳይተላለፍ-------8  ሌላካለይጥቀሱ-------9 | | |  | |  |
| 308 | በአሁኑወቅትጡትእያጠባሽነዉ? | | | አዎ-------1  አይደለም-------2 | | |  | |  |
| 309 | ከላይ 308ላለዉጥያቄመልስሽአዎከሆነ፣መቼለማቆምወሰንሽ? | | | ለማቆም የወሰኑበት እድሜ---------------- | | |  | |  |
| 310 | ከላይ309 ላለዉጥያቄየተጠየቀዉእድሜላይለማቆምየወሰኑበትምክንያቱስምንድንነዉ? | | | ምክንያቶን ይጥቀሱ----------------------- | | |  | |  |
| 311 | ከላይ 308 ላለዉ ጥያቄ መልሱ  አይደለም ከሆነ ጡት ማጥባተን መቼ አቆሙ? | | | ያቆሙበት እድሜ------------------------ | | |  | |  |
| 312 | ከላይ ላለዉ ጥያቄ መልሱ አይደለም ከሆነ ጡት ማጥባት ለምን አቆሙ? | | | ምክንያት፡  የህጻኑመታመም-------1  የእናትመታመም-------2  ከእናትወደልጅበሽተታእንዳይተላለፍ-------3  ሌላካለይጥቀሱ-------4 | | |  | |  |
| 313 | ስለ ልጆት አመጋገብ የምክር አገልግሎት አግኝተዋል? | | | አዎ-------1  አይደለም-------2 | | |  | |  |
| 314 | በቀን ለምን ያህል ጊዜ ልጆትን ይመግባሉ?(ባለፈዉ 24 ሰአት ዉስጥ) | | | --------------------------- | | |  | |  |
| 315 | ባለፉት ሁለት ሳምንታት ዉስጥ ልጅዎት ታሞ ነበር? | | | አዎ-------1  አይደለም-------2 | | |  | |  |
| 316 | አሁን ያሉበት ባህል የአመጋገብ ስርአቱ ላይ ያመጣዉ ተጽእኖ አለ? | | | አዎ--------1  የለም-------2 | | |  | |  |
| 317 | አሁን ያሉበት ልጅዎን የሚመግቡበት ስርአት  ከመፈናቀሎ በፊት ከነበረዉ ጋር ተመሳሳይ ነዉ? | | | አዎ-------1  አይደለም-------2 | | |  | |  |

**መመሪያ፤**ከዚህ በታች የተዘረዘሩትን የምግብ አይነቶች በማንበብ ህጻኑ ከተዘረዘሩትን የምግብ አይነቶች መካከል ከተመገበ አንድ ቁጥርን ያክብቡ፤ ህጻኑ ከተዘረዘሩትን የምግብ አይነቶች መካከል ምንም ካልተመገበ ሁለት ቁጥርን ያክብቡ፡፡

| **ክፍልአራት:ህጻናትየወሰዷቸዉየምግብአይነትመጠይቅ** | | | |
| --- | --- | --- | --- |
| አሁን ልጆ በትላንትናዉ እለት (ባለፈዉ 24 ሰአት ) ዉስጥ ስለተመገበዉ የምግብ አይነት እጠይቆታለሁ፡፡ እባኮ ልጆ በትላንትናዉ እለት(ባለፈዉ 24 ሰአት ) ዉስጥ እቤትም ሆነ ከቤት ዉጪ ስለተመገበዉ የምግብ አይነት ይግለጹልኝ፡፡በትላንትናዉ እለት ጠዋት መጀመሪያ ከተመገበዉ ምግብ ይጀምሩ፡፡ | | | |
| የጥያቄተ.ቁ | **የምግብቡድን** | **ምሳሌዎች** | **ምላሽ** |
| **401** | እህልና የእህል ዘሮች፣ነጭ ሥራ-ሥሮችና ግንዶች | ዳቦ፣ብስኩት፣ኩኪስወይምማንኛዉምከማሽላ፣ዳጉሳእናማንናውምከእነዚህየሚዘጋጅየምገብዓይነት (እነጀራ፣ገንፎ፣ቂጣ ...ወዘተ)ስንዴሩዝበቆሎየተገኘወይምድንችቦዬድንች፣ቦዬ፣ካሳቫ፣እናሌሎችከስራስሮችየሚሰሩምግቦች | አዎ………1  አይደለም..2 |
| 402 | በቫይታሚን ‹‹ኤ›› የበለጸጉ አትክልቶችና  ስራስሮች | ማንጎ (የበሰለ) ፣ፓፓያ ወይም ሌሎች ካሮት፣ ዱባ፣ ቀይሰር እና ስኳር ድንች | አዎ………1  አይደለም..2 |
| 403 | ሌሎች ፍራፍሬ እና አትክልቶች | ቅጠላቸው የሚበላ ደማቅ አረንጓዴ ቅጠል ያላቸው ተክሎች ወይም ሌሎች | አዎ………1  አይደለም..2 |
| 404 | ስጋና የስጋ ውጤቶች | የበሬስጋ፣የአሳማስጋ፣የበግስጋ፣የፍየልስጋ፣የዶሮስጋ እና ትኩስ (ፍረሽ) አሳ ወይም ደረቀ ወይም የባህር አሳ ጉበት፣ኩላሊት፣ልብ፣ደም ወይም ሌላ አካል | አዎ………1  አይደለም..2 |
| 405 | እንቁላል | የዶሮ እንቁላል፣የዳክዬ፣የቆቅ፣የጅግራ ...ወዘተእንቁላል | አዎ………1  አይደለም..2 |
| 406 | ጥራጥሬ | ባቄላ፣ሽምብራ፣አተር፣ኦቾሎኒ እና ከእነዘህ የሚሰሩ ምግቦች በሙሉ | አዎ………1  አይደለም..2 |
| 407 | ወተትና የወተት ተዋጽዖዎች | ወተት፣አይብ፣ዕርጎ፣አሬራ፣ወይምሌላየወተትተዋጽዖ | አዎ………1  አይደለም..2 |
